# Klotho Suppresses Cardiomyocyte Apoptosis in Mice with Stress-Induced Cardiac Injury via Downregulation of Endoplasmic Reticulum Stress

**DOI:** 10.1371/journal.pone.0082968

**Published:** 2013-12-10

**Authors:** Shuang Song, Pan Gao, Hang Xiao, Yan Xu, Lian Yi Si

**Affiliations:** 1 Department of Geriatrics, Southwest Hospital, Third Military Medical University, Chongqing, China; 2 Department of Gynaecology and Obstetrics, Southwest Hospital, Third Military Medical University, Chongqing, China; Tokai University, Japan

## Abstract

Cardiomyocyte apoptosis is a common pathological alteration in heart disease which results in systolic dysfunction or sudden death. Klotho is a novel anti-aging hormone. We tested the effects of klotho on cell apoptosis in isoproterenol-treated cardiomyocytes. In BALB/c mice, cardiac injury was induced by subcutaneous injection of isoproterenol (5mg/kg, for 9days, sc). Klotho (0.01 mg/kg, every other day for 4days, ip) was administered to determine the changes in isoproterenol-induced apoptosis. Mouse heart was harvested at day 2, day 5, and day 9 after isoproterenol injection. Isoproterenol induced cardiac apoptosis and endoplasmic reticulum (ER) stress in a time-dependent manner. However, klotho partly reversed isoproterenol-induced cardiac apoptosis and ER stress. These same effects were observed in cultured cardiomyocytes. Furthermore, the results also showed that SB203580, a p38 inhibitor, and SP600125, a c-Jun NH2-terminal kinase (JNK) inhibitor, reduced cardiomyocyte apoptosis and ER stress, however, klotho suppressed isoproterenol-induced activation of p38 and JNK. Taken together, these results indicated that cardioprotection by klotho was related to the attenuation of ER stress and ER stress-induced apoptosis, at least partly, through suppressing activation of the p38 and JNK pathway.

## Introduction

Regardless of etiology, cardiac remodeling is a common pathological alteration in terms of structure and function in response to various pathogenic stimuli including chronic pressure overload (e.g. essential hypertension), chronic volume overload (e.g. valvular regurgitation), and myocardial infarction[1]. Cardiomyocyte apoptosis plays a key role in the process of remodeling and results in systolic dysfunction or sudden death[2]. Endoplasmic reticulum (ER) is a key participant in cellular apoptosis in response to stress, and disruption of ER homeostasis in response to several pathological stresses activates the ER stress signal and subsequently induces apoptosis when adaptive response fails to alleviate the stress[3–5]. 

Many studies have shown that severe ER stress results in cardiomyocyte apoptosis in vivo and in vitro[3,6]. Inhibitors of ER stress can protect the heart from pathological changes including apoptosis[7,8]. Reports demonstrate that up-regulation of ER chaperone proteins and subsequent transcription induction of pro-apoptosis transcription factor CCAAT/enhancer-binding protein homologous protein (CHOP /GADD153) are involved in ER stress-mediated apoptosis induced by pathogenic stimuli including isoproterenol (ISO) in cultured cardiomyocytes [3,9–11]. CHOP is critical in ER stress-induced apoptosis, and CHOP deficiency inhibits apoptosis[12].The progress of ER stress is initiated by the dissociation of signaling molecules from the abundant chaperone BiP/GRP78 in ER lumen[13]; a prolonged ER stress signal can eventually induce the expression of pro-apoptosis CHOP[14,15]. HSP47 is another ER-resident chaperone, and increased HSP47 also plays an important role in CHOP-mediated apoptosis[16].

Cells regulate intracellular signal pathways in response to exogenous stimuli. Mitogen-activated protein kinases (MAPK), an ubiquitous intracellular signal, consisting of extracellular signal regulated kinase 1/2 (ERK1/2), p38 and c-Jun NH2-terminal kinase (JNK), regulates cellular proliferation, differentiation and apoptosis, and also plays an important regulatory role in ER stress-induced apoptosis[14,17,18]. Therefore, we may assume that endogenous factors which suppress the activation of MAPK and ER stress could decrease cardiomyocyte apoptosis and ameliorate cardiac remodeling.

Klotho exerts anti-aging effects, and klotho deficiency results in multiple age-related disorders closely resembling human aging, including atherosclerosis, ectopic calcification and shortened lifespan[19]. The klotho gene encodes a single-pass transmembrane protein predominantly expressed in renal tissue. In addition, the extracellular domain of klotho is shed and released into circulation known as secreted klotho[19,20]. Accumulative evidence indicates that secreted klotho exerts resistance to oxidative stress and inflammation via regulation of intracellular signal pathways including p38 MAPK [21,22]. In addition, klotho-deficient mice develop exaggerated remodeling and apoptosis in response to ISO treatment, suggesting a cardioprotective effect by klotho[23]. However, the link between klotho and the ER stress-driven apoptotic signal has not been elucidated.

Cardiac remodeling induced by ISO injection is a well-established model[24]. We designed the present study to determine whether klotho alleviated ISO-induced cardiac remodeling. We also investigated the protective effect of klotho on ER stress and subsequent apoptosis regulated by the MAPK signaling pathway.

## Materials and Methods

### Reagents

Recombinant mouse klotho protein was purchased from R&D system (Minneapolis, MN). Isoproterenol was purchased from Sigma (St Louis, MO). The antibodies for CHOP/GADD153, GRP78 and HSP47 were purchased from Santa Cruz Biotechnology (Santa Cruz, CA). The antibodies for p38, p-p38 (Ser473), ERK1/2, p-ERK1/2 (Thr202/Tyr204), CHOP and beta-actin, PD98059, SP600125 and SB203580 were acquired from Cell Signaling Technology (Boston, MA). TUNEL assay in situ apoptosis detection kit was purchased from Roche (Basel, Swit). All other chemicals were reagents of molecular biology grade obtained from standard commercial sources.

### Ethics statement

Animal experiments were approved by Laboratory Animal Welfare and Ethic Committee of the Third Military Medical University (SCXK-PLA-2007015), in accordance with the US National Institutes of Health (NIH, 8th edition, 2011). 

### Animal experiments

Male 6- to 8-week-old BALB/c mice (20-25g) were randomly assigned to one of the following groups. The **CON** group (n=18) received subcutaneous injections of saline (0.1 mL, per day for 9 days) and intraperitoneal injections of saline (0.1 mL, every other day for 4 days); the **ISO** group (n=17) received subcutaneous injections of ISO (5 mg/kg, dissolved in 0.1 mL saline, per day for 9 days) and intraperitoneal injections of saline (0.1 mL, every other day for 4 days); the **ISO+KL** group (n=18) received intraperitoneal injections of recombinant klotho protein (0.01 mg/kg, dissolved in 0.1 mL saline, every other day for 4days) plus the daily injections of ISO as above[25,26]. The mice were euthanized at day2, day5 and day9 after ISO treatment. All efforts were made to ameliorate suffering of animals. 

### Tissue preparation and histological analyses

The hearts were carefully excised, washed in cold PBS and freed of connective tissue at day 2, day 5 and day 9 respectively. Each heart was weighed (HW), and HW was normalized by body weigh (HW/BW Ratio). Each heart was cut into six blocks, three blocks were stored in liquid nitrogen for Real-time PCR and western blot assay. The remaining blocks from LV were fixed in 4% formaldehyde for 24 hours and embedded in paraffin. Serial sections of 4 um were stained with hematoxylin/eosin (HE) for morphologic analysis. For collagen quantification, serial sections were also stained with Masson’s trichrome. Images were captured with a Leica microscope (Leica DMI3000 B). The collagen volume fraction (CVF) was determined (the ratio of interstitial collagen area to myocardial area) by Image-Pro Plus 6.0 ( IPP 6.0, Media Cybernetics, MD, USA) for an average of 10 micrographs[27].

### Terminal transferase-mediated dUTP nick-end labeling (TUNEL) assays

For the analysis of cardiac apoptosis, TUNEL assay was performed according to the specifications for in situ apoptosis detection kit. Micrographs were obtained using a Leica microscope (Leica DMI3000 B). To determine the apoptosis rate, 10 random fields from each section were quantified in a blinded manner. The total cell numbers in each section were about 2000. Three sections per group were used[28].

### In situ detection of ROS production

Oxidative stress in myocardium was determined by detecting the ROS production in situ by dihydroethidium (DHE, Biotype Biotechnology). Fluorescence images were obtained using a fluorescence microscope (Olympus, Japan) equipped with a rhodamine filter[29].

### Immunohistochemistry

Paraffin-embedded cardiac sections were dewaxed, and then washed in Tris-buffered saline (TBS) containing 3% bovine serum albumin (BSA). Endogenous peroxidase activity was quenched by 3% H_2_O_2_ in methanol. These sections were incubated with anti-GRP78, anti-HSP47 and anti-CHOP (diluted 1:100), and developed with corresponding kit. The cell nuclei were counterstained with hematoxylin. The optical densities were quantified by IPP 6.0 at 10 randomly-selected fields per sample. Three sections per group were used.

### Quantitative real-time PCR analysis

Total RNA was extracted from frozen myocardial tissues using Trizol. Ultramicro-ultraviolet spectrophotometry (Beckman) was used to measure the concentration and purity. A ReverTra Ace® qPCR RT Kit was used for reverse transcription. A SYBR® green real-time PCR Master Mix-Plu Kit was used to perform the Real-time PCR analysis using the following primers. GRP78, 5′-GCG TGT GTG TGA GAC CAG AAC CG-3′ (up), 5′-TGG TTG CTT GTC GCT GGG CA-3′ (down); HSP47, 5′-GGC GAT TTG GGG TTG CGC ATT-3′ (up), 5′-GCT CTG CCA GTG TGG TCG CC-3′ (down); CHOP, 5′-GCC GGA ACC TGA GGA GAG AGT GT-3′ (up), 5′-ACT CAG CTG CCA TGA CTG CAC G-3′ (down); Beta-actin, 5′-GTG GGC CGC CCT AGG CAC CA-3′ (up), 5′-CGG TTG GCC TTA GGG TTC AGA GGG-3′ (down). On the basis of the Ct values of the target gene and internal control gene, the 2^−ΔΔCt^ method was used to calculate the relative expression levels of GRP78, HSP47 and CHOP in each group.

### Western blot analysis

Proteins were separated by 10% SDS-PAGE and transferred onto a nitrocellulose membrane. The membrane was washed in TBS-T (0.05% Tween-20 in TBS), blocked with 5% BSA in TBS-T, and incubated with antibodies against phospho-p38 (1:800), phospho-ERK1/2 (1:1200), total-p38 (1:800), total-ERK1/2 (1:1200), GRP78 (1:500), HSP47 (1:500), CHOP (1:1000) and beta-actin (1:1200). Following three washes with TBS-T, the membrane was incubated with HRP-conjugated secondary antibodies and assessed by enhanced chemiluminescence detection reagent and autoradiography (Kodak, Rochester, NY, USA). Image was analyzed using Image lab software.

### Cell culture

It has been reported that the H9c2 cell line (embryonic rat heart derived) is an ideal model for in vitro studies of cardiac injury as it shows almost identical pathological responses to those observed in primary cardiomyocytes[30]. H9c2 cells were purchased from the Cell Bank of Chinese Academy of Science (Shanghai, China), and grown in DMEM containing 10% fetal bovine serum (FBS).

### Cell apoptosis assay

To determine the effect of different treatments on apoptosis, H9c2 cells were plated in 6-well plates and treated with arranged stimuli. Apoptotic cells were measured using a PI/Annexin V-FITC kit on a FACScan flow cytometer (Becton Dickinson, Mountain View, CA).

### Intracellular ROS production

H9c2 cells were plated in 6-well plates. Treatment was terminated with ice-cold PBS and then incubated with 5uM DCF-DA. The DCF fluorescence intensity was measured on a FACScan flow cytometer using 485 nm excitation and 535 nm emission filters. 

### Statistical analysis

SPSS (version 13.0) was used for statistical analyses. All experiments were repeated at least three times. Data are the mean±SEM. Groups of data were compared with ANOVA, followed by Tukey’s multiple comparison tests. Data that did not follow a normal distribution were analysed with the Mann–Whitney test or Student’s t-test after log normalization. P <0.05 was considered significant. 

## Results

### 1: Klotho inhibited ISO-induced pathological changes in mouse heart

**Figure 1:**
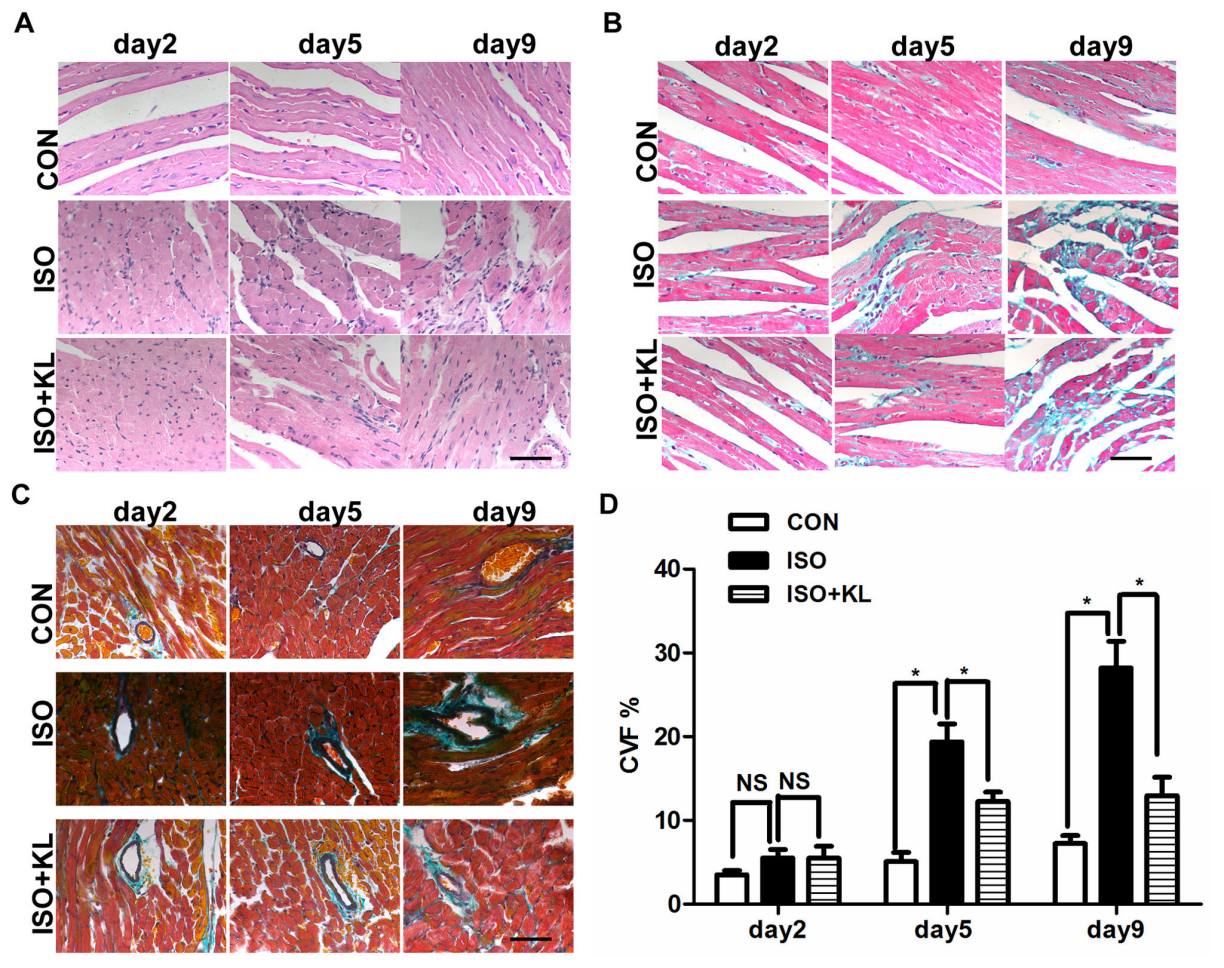
Klotho reduced ISO-induced histological changes in mouse heart. (A) Representative images of HE staining of mouse heart in CON, ISO and ISO+KL groups for 2, 5 and 9 days. Scale bar=50 um, 400×. (B) Representative images of Masson trichrome staining of LV sections in CON, ISO and ISO+KL groups for 2, 5 and 9 days. Blue: Fibrous collagen; Red: myocyte. Scale bar=50 um, 400×. (C) Representative images of Masson trichrome staining of perivascular collagen in CON, ISO and ISO+KL groups for 2, 5 and 9 days. Scale bar=50 um, 400×. (D) Quantitative plot of LV collagen volume fraction (CVF%) indicated by blue area as opposed to the red myocardium. Data are mean±SEM, n=5. * P<0.05 between two compared groups; NS, no significance.

We detected the overall circulating level of klotho in serum and found that it was significantly increased after administration of recombinant klotho (Figure S1). To determine whether klotho could inhibit pathological changes in mouse with ISO-induced cardiac injury, a mouse model of cardiac remodeling was induced following subcutaneous injection of ISO for 9 days. We observed cardiac histological difference between ISO group and ISO+KL group at day 2, day 5 and day 9. In the control group, myocardial fibers were arranged regularly. However, histological sections of mouse heart showed disordered arrangement of myocardial fibers, abundant fibroblastic hyperplasia evidentiated by an increase in the nuclei density and a few of focal mononuclear cell infiltration after ISO injection, indicating serious pathological changes in ISO group (Figure 1A). However, klotho ameliorated these changes during the same period, suggesting a cardioprotective effect by klotho (Figure 1A). Masson trichrome staining showed significantly increased interstitial and perivascular fibrosis in ISO group from day 5 compared with CON group, and klotho partly reduced cardiac fibrosis from day 5 compared with ISO group (Figure 1B, C, D). These results suggested that klotho inhibited ISO-induced structural changes in mouse heart. 

### 2: Klotho reduced ISO-induced apoptosis in mouse heart

**Figure 2:**
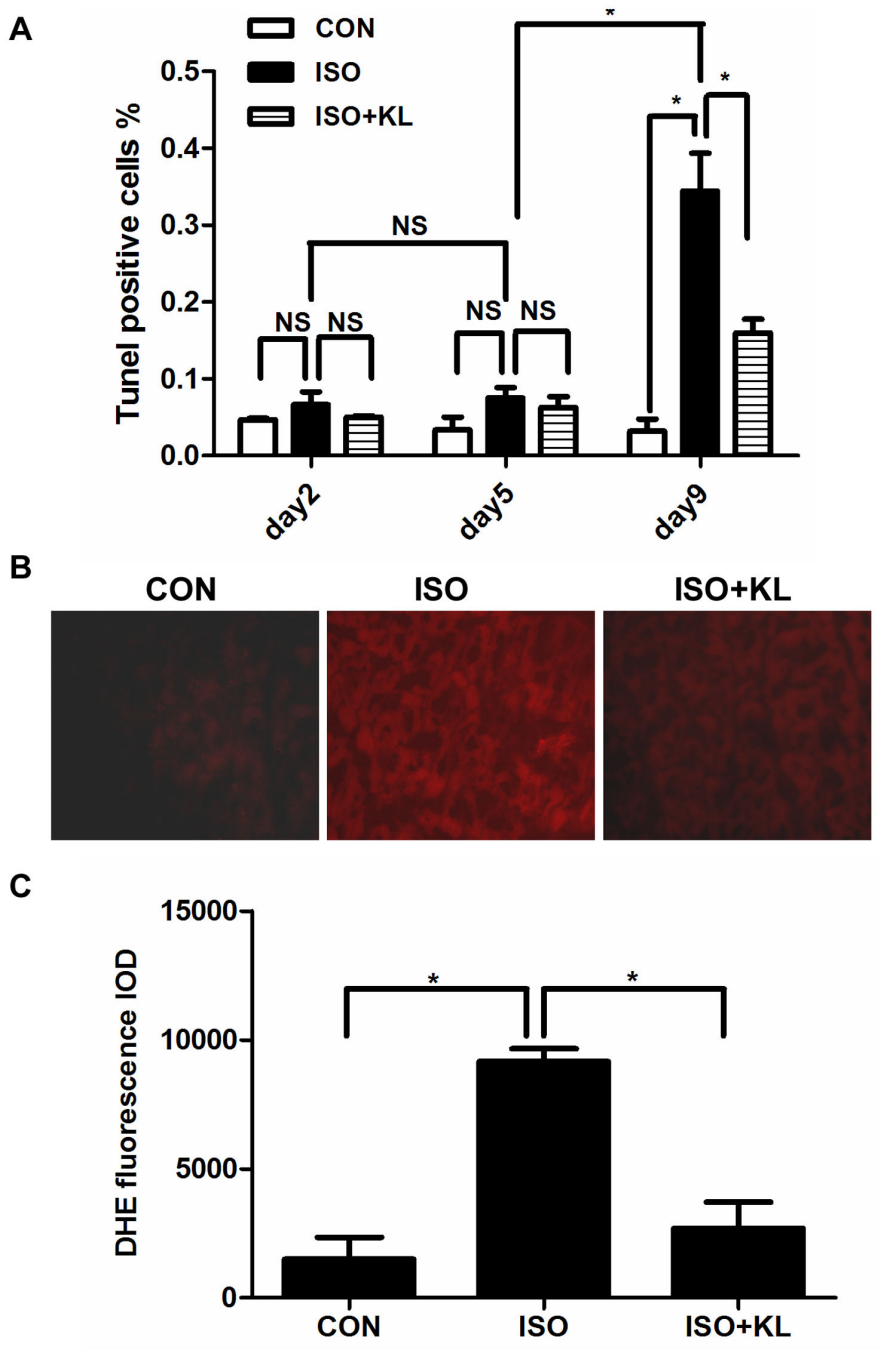
Klotho reduced ISO-induced apoptosis and ROS production. (A) Quantitative plot of TUNEL positive cells in CON, ISO and ISO+KL groups for 2, 5, 9 days. Apoptosis rate was determined by the ratio of apoptotic nuclei to total nuclei. The average total cells for each section are about 2000. Data are mean±SEM, n=3. * P<0.05 between two compared groups; NS, no significance. (B) Representative images of Dihydroethidium (DHE) staining of LV sections in CON, ISO and ISO+KL groups for 9 days . Red fluorescence represented the ROS production.200×. (C) Quantitative plot of relative DHE fluorescence intensities normalized by CON. Data are mean±SEM, n=3. * P<0.05 between two compared groups.

Apoptosis plays a major role in myocardial remodeling and causes cardiac dysfunction via cardiomyocytes loss. In addition, oxidative stress is an important regulator of apoptosis. So we detected the effects of klotho on apoptosis and ROS production in mouse heart. Results showed that ISO significantly increased the percentage of TUNEL-positive cells compared with CON group at day 9. (Figure 2A, Figure S2).Administration of klotho significantly reduced the percentage of TUNEL-positive cells at day 9 although it did not reduce it at day 2 and day 5 (Figure 2A, Figure S2), indicating the chronic supplement of klotho could inhibit apoptosis. In accordance with these results, ROS were significantly stimulated in ISO group, but were abated in ISO+KL group (Figure 2B,C). 

### 3: Klotho inhibited the ER stress-induced apoptotic signal in mouse heart

**Figure 3:**
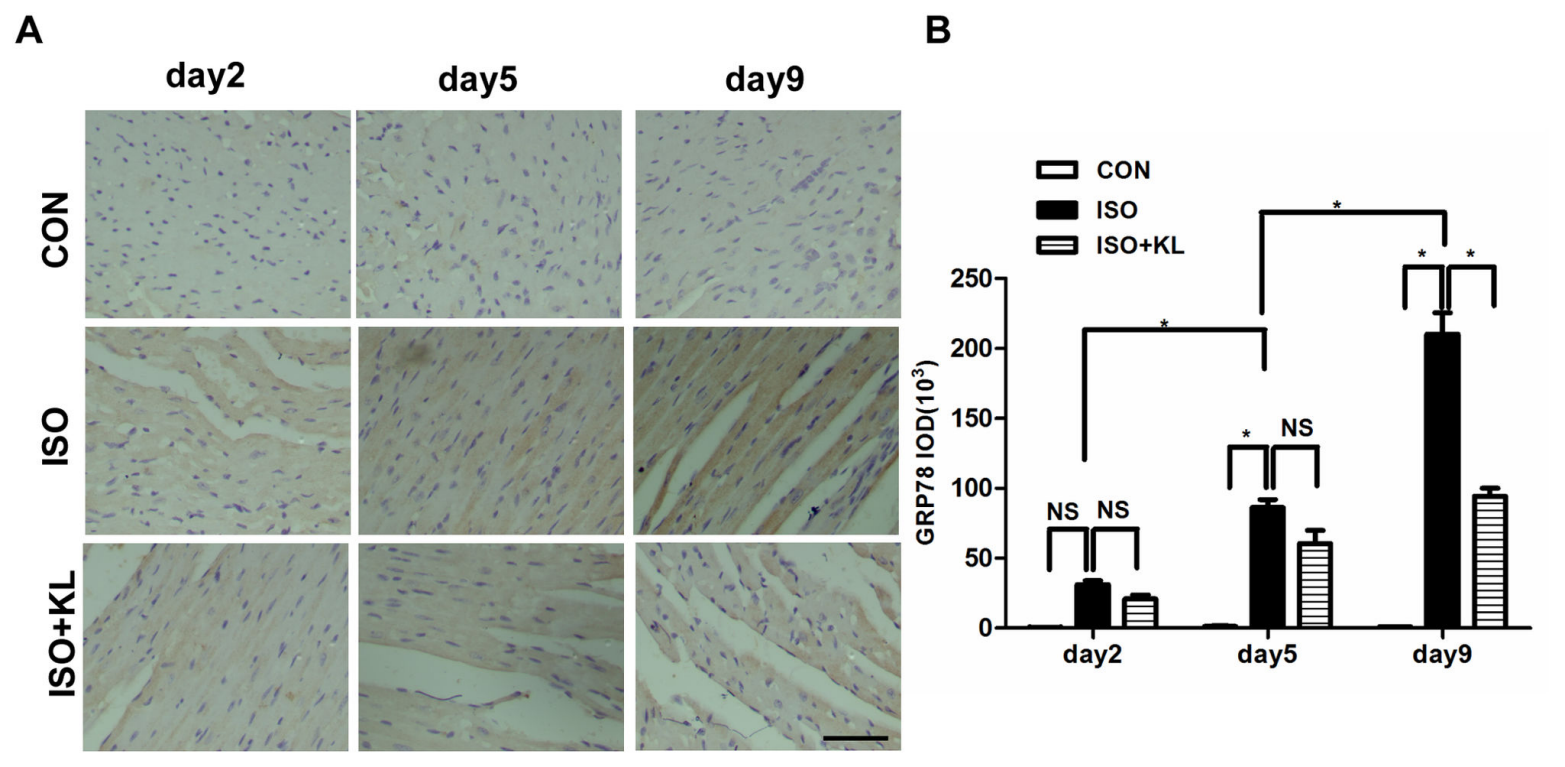
Klotho decreased the expression of GRP78 in mouse heart. (A) Representative images of immunohistochemical staining for GRP78, in CON, ISO and ISO+KL groups for 2, 5 and 9 days. Scale bar=50 um, 400×.(B) Quantitative plot of average expression of GRP78. The expression level of GRP78 in the ISO+KL group was significantly lower than in the ISO group at day 9. Data are mean±SEM, n=3-4. * P<0.05 between two compared groups; NS, no significance.

**Figure 4:**
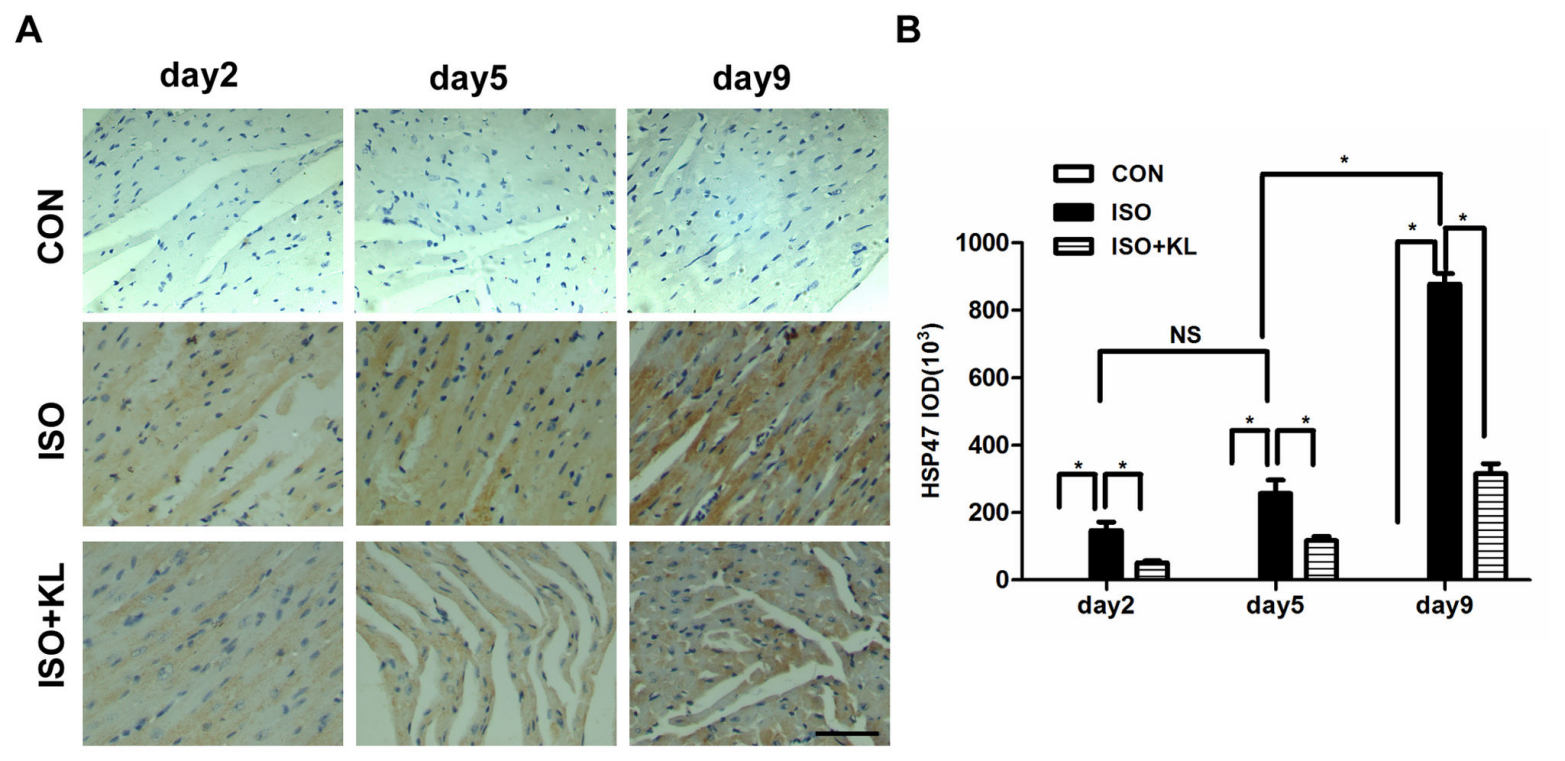
Klotho decreased the expression of HSP47 in mouse heart. (A) Representative images of immunohistochemical staining for HSP47 in CON, ISO and ISO+KL groups for 2, 5 and 9 days. Scale bar=50 um, 400×. (B) Quantitative plot of average expression of HSP47. The expression level of HSP47 in the ISO+KL group was significantly lower than in the ISO group at day 2, 5, 9. Data are mean±SEM, n=5. * P<0.05 between two compared groups; NS, no significance.

**Figure 5:**
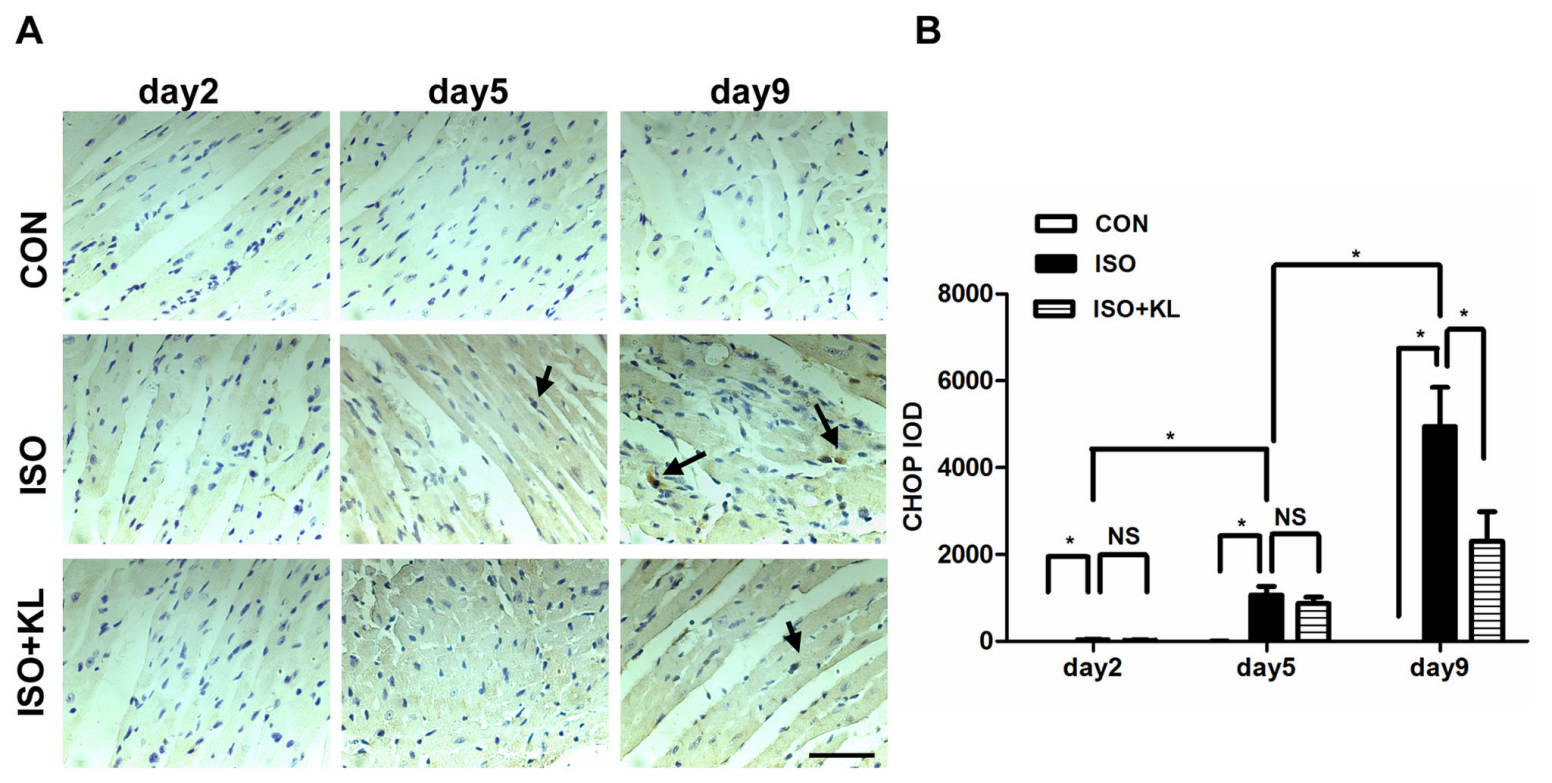
Klotho decreased the expression of CHOP in mouse heart. (A) Representative images of immunohistochemical staining for CHOP in CON, ISO and ISO+KL groups for 2, 5 and 9 days. Scale bar=50 um, 400×. Black arrows represent the positive staining of CHOP expressed in nuclei. (B) Quantitative plot of average expression of CHOP. The expression level of CHOP in the ISO+KL group was significantly lower than in the ISO group at day 9. Data are mean±SEM, n=5 . * P<0.05 between two compared groups; NS, no significance.

**Figure 6:**
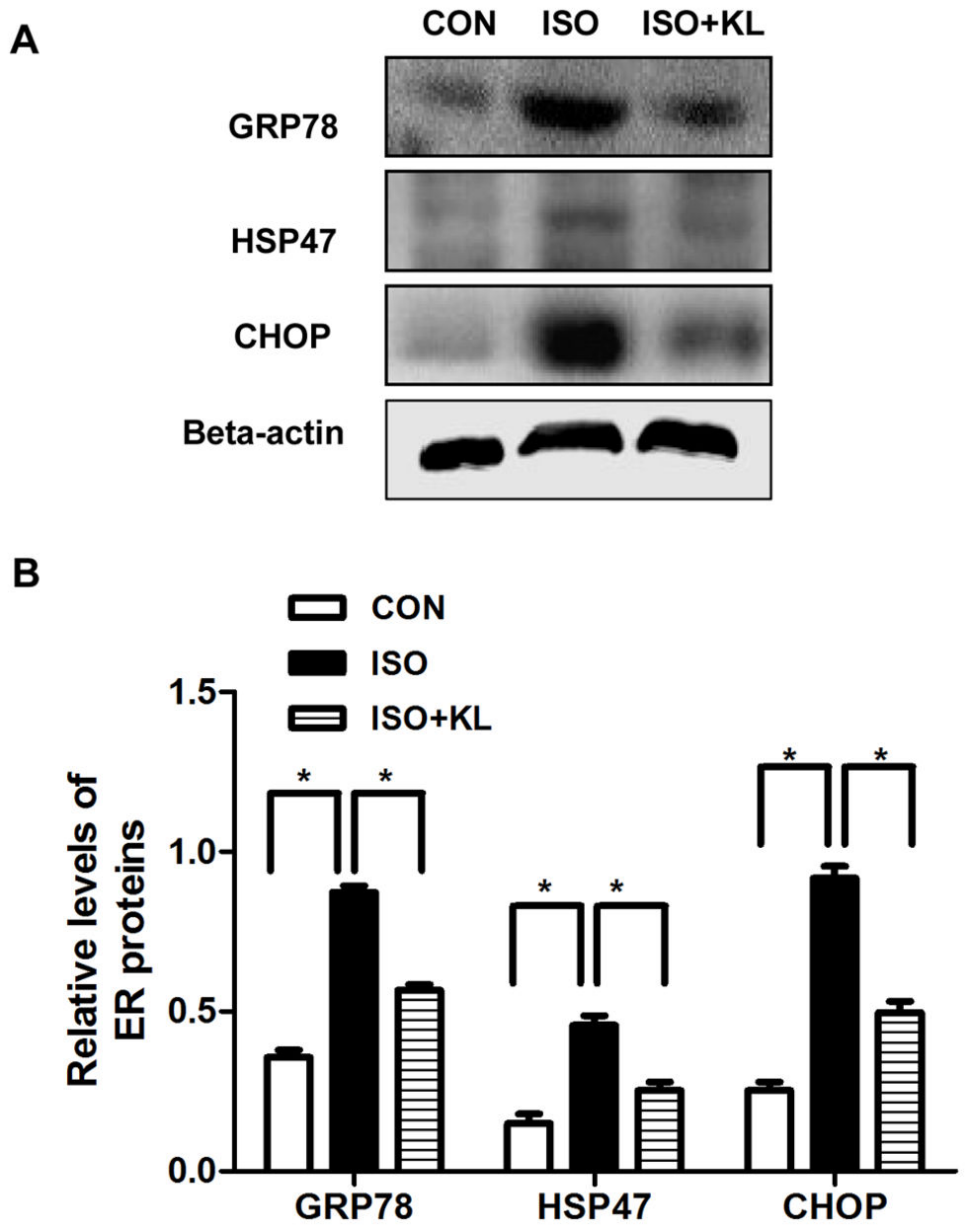
Klotho decreased the expression of ISO-induced ER chaperone proteins in mouse heart. (A) Representative images of western blotting of GRP78, HSP47, CHOP and beta-actin in CON, ISO and ISO+KL groups for 9 days. (B) Demonstration of the expression level of GRP78, HSP47 and CHOP in CON, ISO and ISO+KL groups. Data are mean±SEM, n=3. * P<0.05 between two compared groups; NS, no significance.

**Figure 7:**
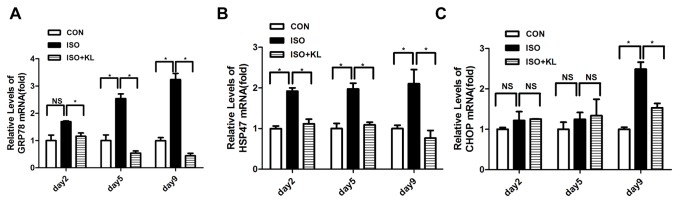
Klotho decreased the mRNA of ER stress-related proteins in ISO-stimulated mouse heart. (A,B,C) Quantitative plots of GRP78, HSP47 and CHOP mRNA in CON, ISO and ISO+KL groups for 2, 5 and 9 days. Klotho decreased the GRP78 and HSP47 mRNA from day2 to day9, and it significantly reduced CHOP mRNA at day 9. Data are mean±SEM, n=3. * P<0.05 between two compared groups; NS, no significance.

To further determine the effect of klotho on the apoptotic signal, we detected the expression of ER stress biomarker (GRP78, HSP47) and ER stress-related pro-apoptosis factor (CHOP) in different groups. Immunohistochemical staining for GRP78 showed that ISO statistically increased the expression of GRP78 from day 5 compared with CON group. Klotho did not significantly decrease the expression of GRP78 at earlier stage (day2,day5), but it succeed at day9 (Figure 3A,B). Immunohistochemical staining for HSP47 showed that the expression of HSP47 in ISO group was statistically higher than in CON group from day2 to day9. Klotho markedly decreased the expression of HSP47 from day2 to day9 (Figure 4A,B). Results also showed that the expression of CHOP in ISO group was higher than in CON group from day2 to day9. Importantly, klotho statistically decreased the expression of CHOP at day 9 (Figure 5A, B). Western blotting for GRP78, HSP47, and CHOP also showed identical results (Figure 6A, B). Real-time PCR for GRP78, HSP47, and CHOP showed dramatic increases in mRNA levels of ER stress markers in ISO group, but it was partially attenuated in ISO+KL group (Figure 7A, B, C). Thus, ISO-induced sustained ER stress and apoptosis in mouse heart were partly prevented by klotho. 

### 4: Klotho reduced ISO-induced apoptosis in H9c2 cells

**Figure 8:**
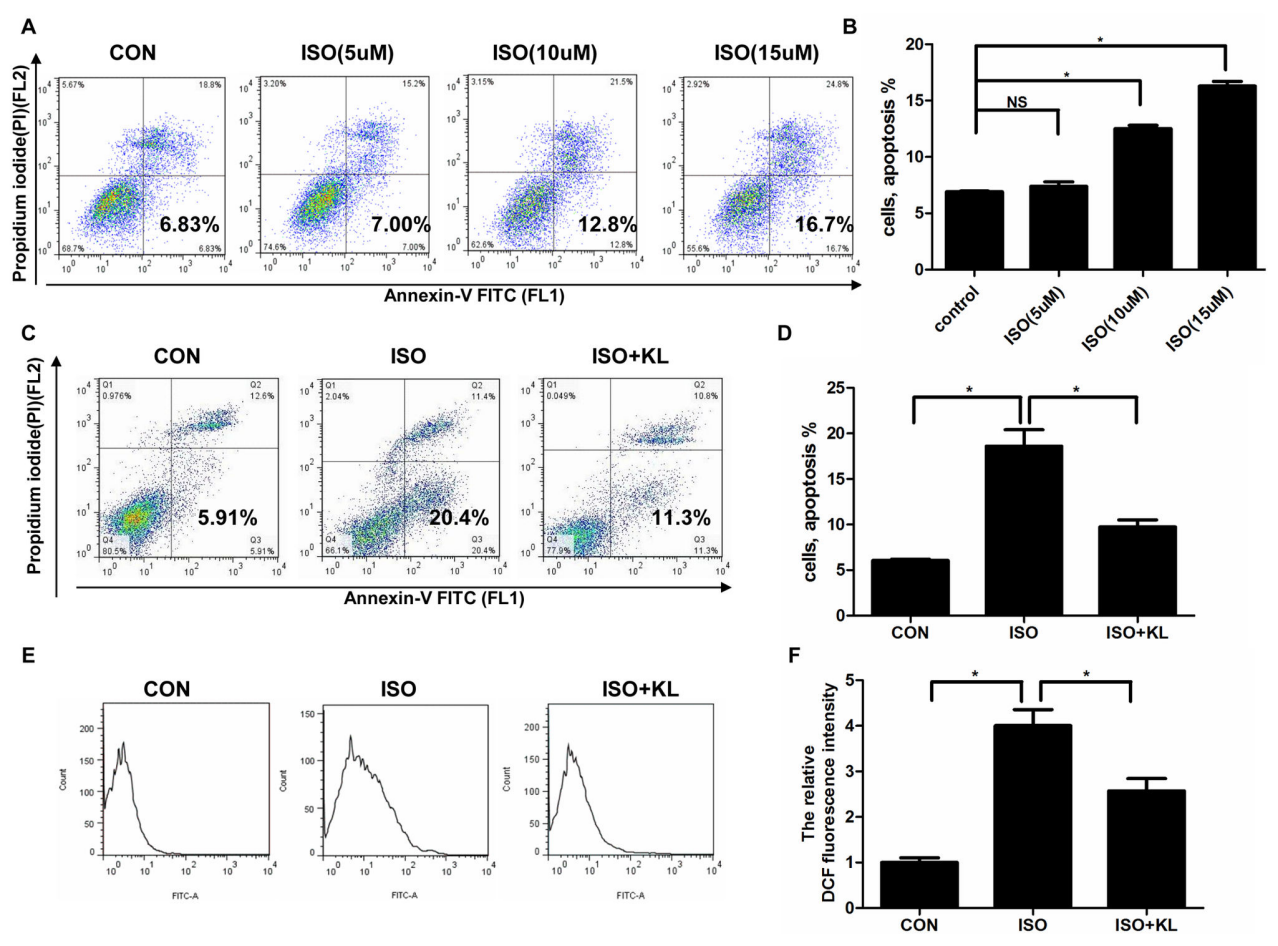
Klotho reduced ISO-induced apoptosis and ROS production in H9c2 cells. (A) Representative images of apoptosis assay in H9c2 cells treated by different concentrations of ISO. (B) Demonstration of apoptosis rate.(C) Representative images of apoptosis assay in H9c2 cells treated by ISO and/or klotho. (D) Demonstration of apoptosis rate. Data are mean±SEM, n=5. * P<0.05 between two compared groups; NS, no significance. (E) Representative images of DCF fluorescence signal assessed by FCS Express. (F) Demonstration of the relative DCF fluorescence intensity normalized by CON. Data are mean±SEM. n=3 * P<0.05 between two compared groups; NS, no significance.

The above in vivo studies clearly indicated an inhibitory effect by klotho on ISO-induced cardiac apoptosis. In vitro study showed that there was a dose-dependent increase in cell apoptosis following exposure of H9c2 cells to ISO (Figure 8A,B). As a study demonstrated the induction of apoptotic cell death by ISO at the concentration of 10 uM in H9c2 cells[31], we selected 10 uM in our subsequent study. To determine the effect of klotho on apoptosis in cultured cells, we treated H9c2 cells with ISO and/or klotho. Results showed that klotho statistically reduced ISO-induced apoptosis (Figure 8C, D). We also found that ISO stimulated ROS production, which were partly prevented by klotho (Figure 8E,F). These results indicated the klotho’s anti-apoptosis property.

### 5: Klotho inhibited apoptosis through the amelioration of ER stress in H9c2 cells

**Figure 9:**
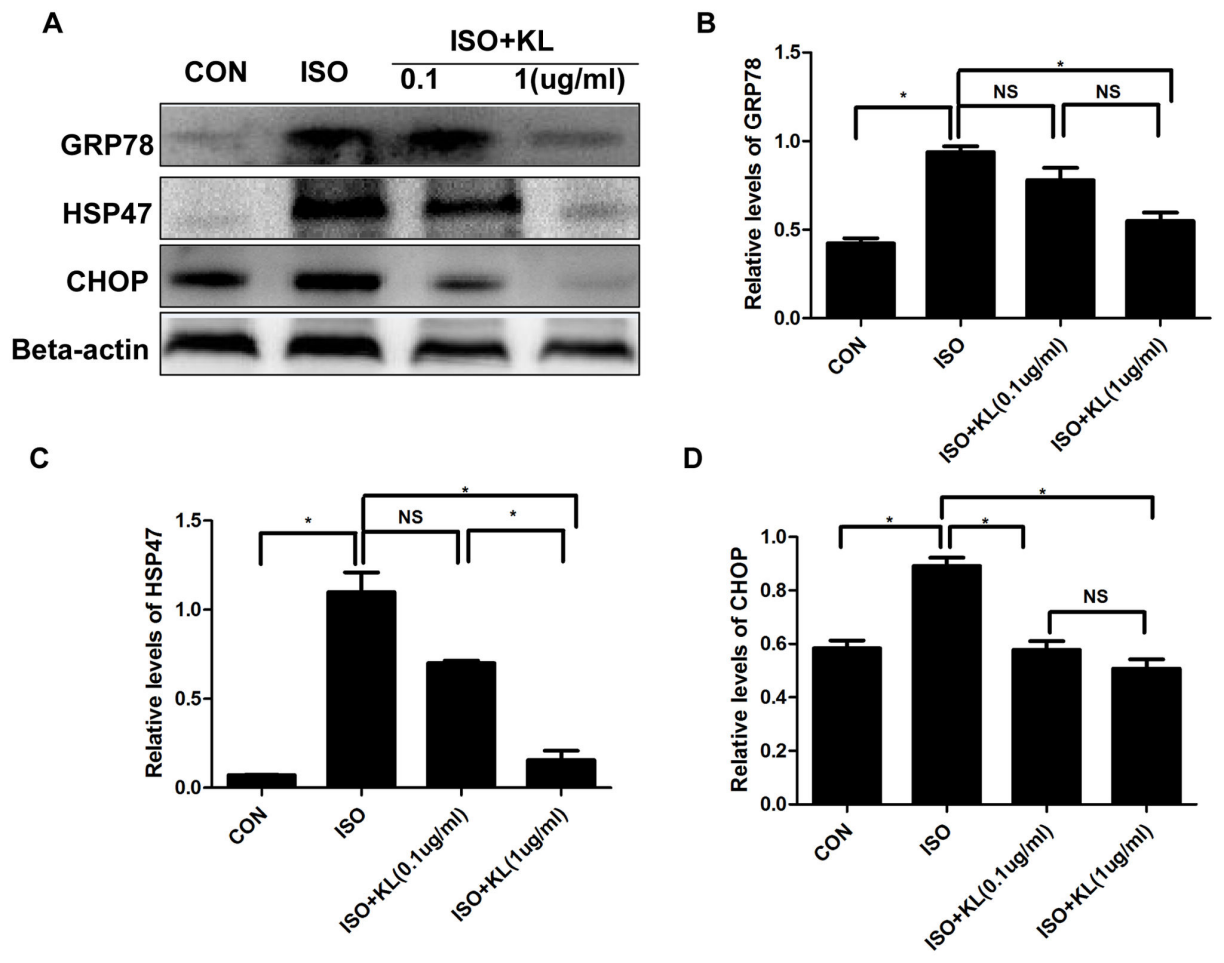
Klotho ameliorated ISO-induced ER stress in H9c2 cells. (A) The representative images of western blotting of GRP78, HSP47, CHOP and beta-actin in CON, ISO, ISO+KL(0.1 ug/ml) and ISO+KL( 1 ug/ml) groups.(B,C,D) Demonstration of the expression level of GRP78(B), HSP47(C) and CHOP(D) in CON, ISO, ISO+KL(0.1 ug/ml) and ISO+KL( 1 ug/ml) groups. Data are mean±SEM, n=3. * P<0.05 between two compared groups; NS, no significance.

In the present study, ISO significantly increased the expression of GRP78, HSP47 and CHOP, suggesting the activation of ER stress and ER stress-related apoptotic signal (Figure 9A, B, C, D). Pretreatment of H9c2 cells with 1ug/ml of klotho before exposure to ISO effectively ameliorated these changes although 0.1ug/ml of klotho did not significantly reduce the levels of GRP78 and HSP47 (Figure 9A, B, C). These results indicated the anti-apoptosis character of klotho was dependent on the inhibition of ER stress.

### 6: The MAPK pathway was involved in the anti-apoptosis effect of klotho

**Figure 10:**
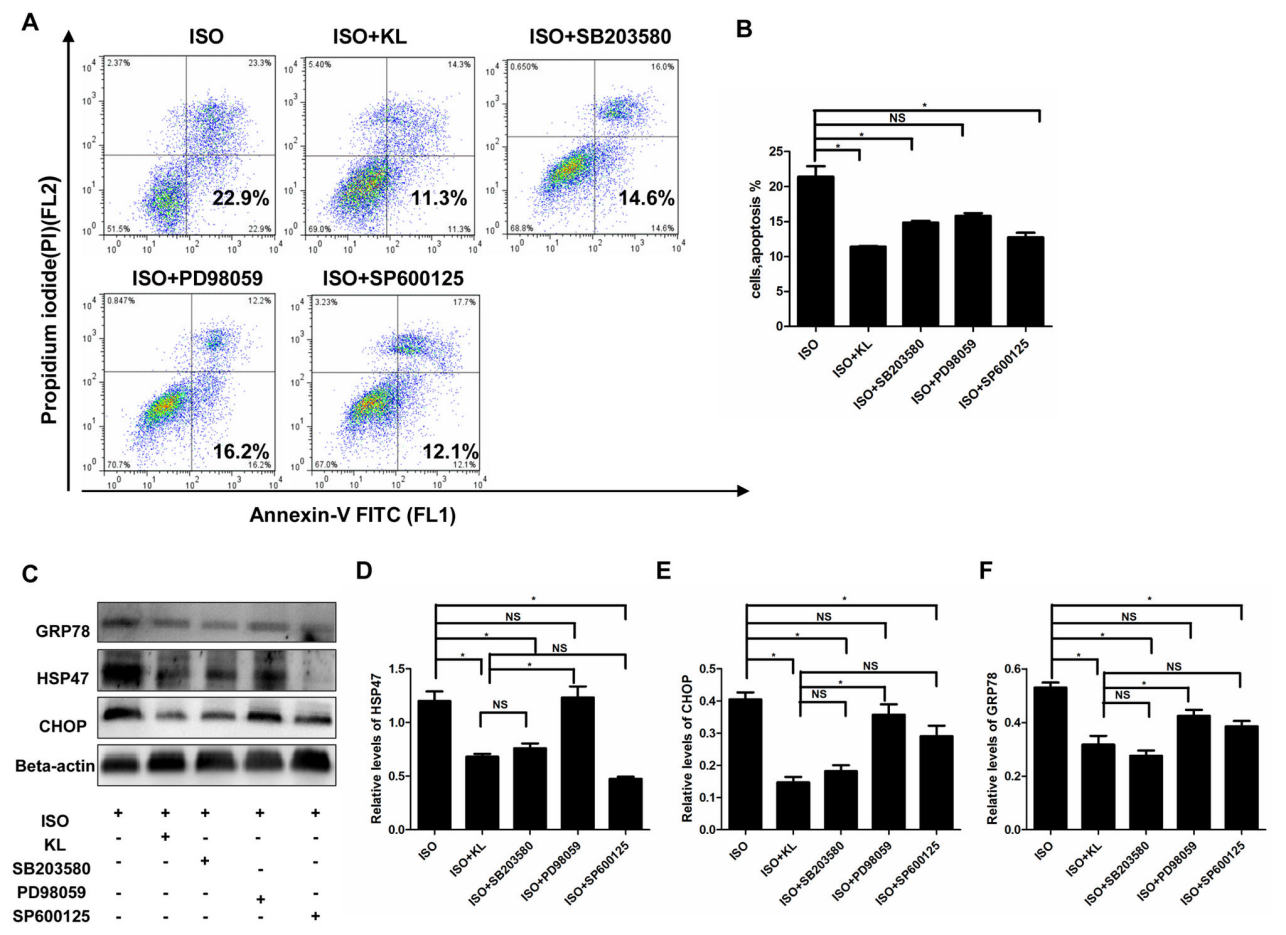
Anti-apoptosis effect of klotho was dependent on MAPK. (A) Representative images of apoptosis assay assessed by flow cytometry in H9c2 cells treated by ISO, ISO+KL, ISO+SB203580, ISO+PD98059, and ISO+SP600125. (B) Demonstration of apoptosis rate in ISO, ISO+KL, ISO+SB203580, ISO+PD98059, and ISO+SP600125 groups. (C) Representative images of western blotting of GRP78, HSP47, CHOP and beta-actin in ISO, ISO+KL, ISO+SB203580, ISO+PD98059, and ISO+SP600125 groups. (D,E,F) Demonstration of the expression level of GRP78(D), HSP47(E) and CHOP(F) in ISO, ISO+KL, ISO+SB203580, ISO+PD98059, and ISO+SP600125 groups. Data are mean±SEM, n=3. * P<0.05 between two compared groups; NS, no significance.

In accordance with the previous results, klotho significantly reduced ISO-induce apoptosis. Pretreatment of cells with SB203580 or SP600125 also induced a marked reduction in apoptosis (Figure 10A, B). However, PD98059 did not statistically inhibit apoptosis. In addition, the levels of GRP78, HSP47 and CHOP were significantly decreased by klotho, SB203580 and SP600125, but not by PD98059 (Figure 10 C, D, E, F).These results suggested that klotho ameliorated ER stress and ER stress-induced apoptosis through inhibition of the p38 and JNK pathway.

**Figure 11:**
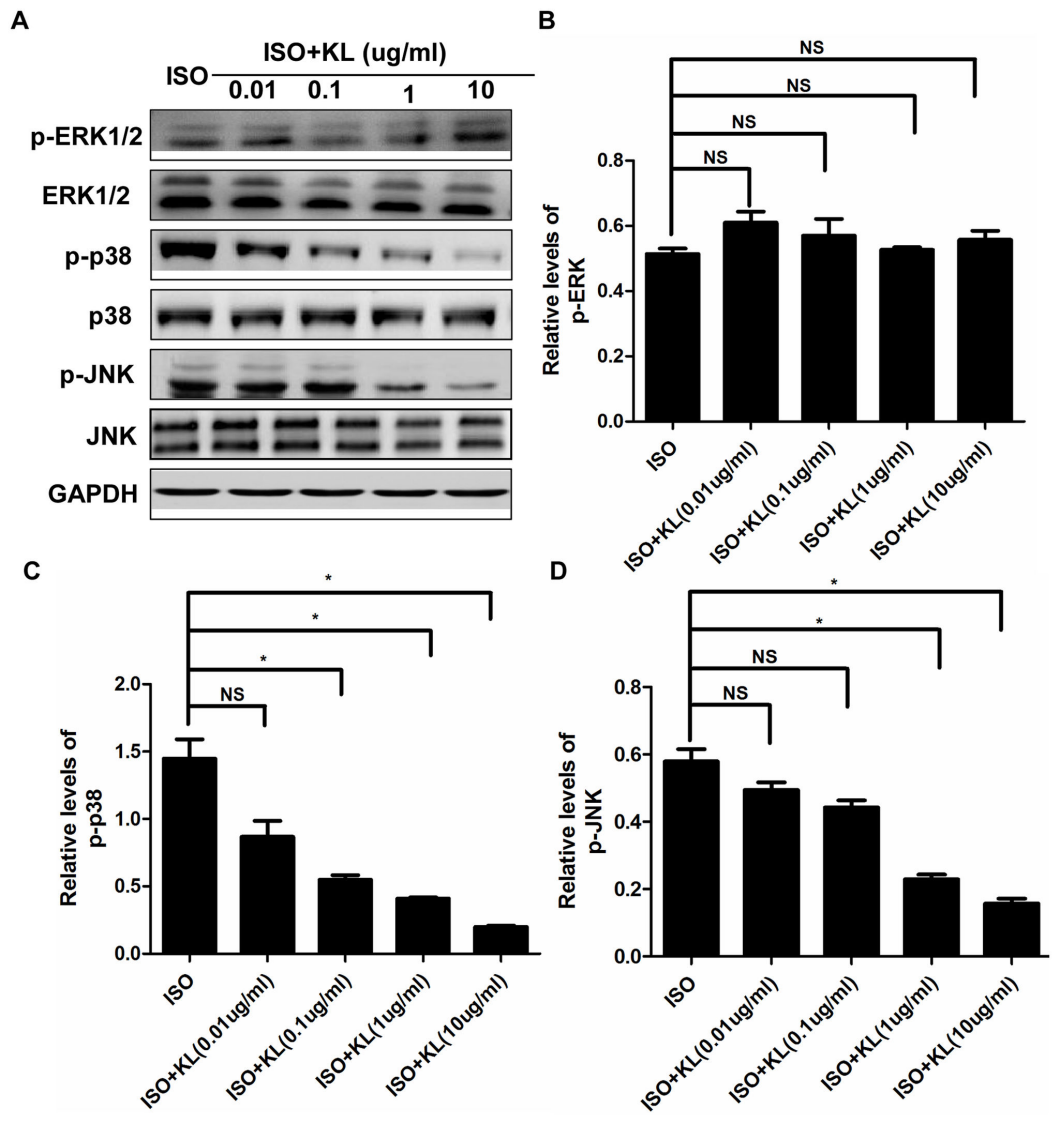
Klotho regulated the phosphorylation of MAPK. (A) Representative images of western blotting of p-ERK1/2, ERK1/2, p-p38, p38, p-JNK, JNK and GAPDH in H9c2 cells treated by ISO and/or different concentrations of klotho (0.01, 0.1, 1, 10ug/ml). (B,C,D) Demonstration of the phosphorylation of p-ERK1/2(B), p-p38(C) and p-JNK(D) in ISO, ISO+KL(0.01ug/ml), ISO+KL(0.1ug/ml), ISO+KL(1ug/ml), ISO+KL(10ug/ml) groups. Data are mean±SEM, n=3. * P<0.05 between two compared groups; NS, no significance.

We further determined the direct effect of klotho on MAPK. Results showed that ISO activated the phosphorylation of MAPK in H9c2 cells (Figure S3A, B, C, D), and addition of klotho (0.01-10ug/ml) did not statistically affect the phosphorylation of ERK1/2 (Figure 11 A, B), but it significantly stimulated the phosphorylation of p38 and JNK at 0.1ug/ml and 1ug/ml respectively (Figure 11A, C, D). These results indicated anti-apoptosis effect by klotho was partly dependent on suppression of the p38 and JNK pathway. 

## Discussion

The protective effect of klotho observed in this study included amelioration of the structural changes in mouse heart. More importantly, klotho inhibited cell apoptosis in vivo and in vitro, which was, at least partly, attributed to suppression of the ER stress-induced apoptotic signal. However, specific p38 and JNK inhibitors (SB203580 and SP600125) efficiently suppressed ER stress and subsequent apoptosis. Moreover, klotho partly inhibited phosphorylation of the p38 and JNK pathway in H9c2 cells. so we concluded that klotho inhibited ISO-induced ER stress and apoptotic signal through suppression of the P38 and JNK pathway, and finally improved cardiac pathological changes.

H9c2 cells display morphological characteristics similar to those of immature embryonic cardiocytes, and preserve to a large extent the signaling pathways found in adult cardiac cells[32]. Many studies also showed that H9c2 cell line had been extensively used to investigate the pathological process of cardiac hypertrophy and apoptosis. Furthermore, ISO induced the same ER stress in H9c2 cells as in primary cultured cardiomyocytes[3,9,31]. So our study used H9c2 cells as a cardiomyocyte apoptosis model to investigate the effect of klotho on ER stress-induced apoptosis.

Klotho was reported to resist oxidative stress at both organism and cellular level in mammals, which might potentially contribute to its anti-aging property[33].Recently, many studies showed that klotho reduced apoptosis and oxidative stress via regulation of the signal pathways in endothelial cells [34]. Clinical studies also showed that a decreased level of serum klotho might be an early biomarker for chronic kidney disease-induced cardiovascular disorder[35].However, studies on the cardioprotection of klotho are limited. This study demonstrated that klotho played a protective role in ISO-induced cardiac pathologies, which was supported by another study[23]. Most notably, we also found that klotho inhibited ISO-induced cardiomyocyte apoptosis, which was potentially contributed to amelioration of the ER stress-induced apoptotic signal. So it is reasonable to conclude that amelioration of ER stress might be another new and important effect of klotho.

Increasing evidence has shown that ER is recognized as an important organelle which participates in the apoptotic pathway. ER stress is a common denominator which mediates damaging effect especially apoptosis [10]. Induction of ER chaperone proteins is an early adoptive response to ER stress, and is also a mark of the severity of stress. In this study, we found that klotho down-regulated ER chaperone proteins in response to ISO. In accordance with our finding, it was reported that downregulation of ER chaperone proteins was beneficial in heart apoptosis [36]. Paradoxically, induction of GRP78 by gene transfer or pharmacological agents protected cardiomyocytes from hypoxic injury [37]. The reason for these differences may be that the function of GRP78 depends on the severity of the stress or the difference in stimulatory conditions. In the early stage of ER stress, compensatory induction of GRP78 ameliorated ER stress and inhibited apoptosis. But prolonged or excessive ER stress induced GRP78 and resulted in apoptosis. HSP47 is also a ER chaperone, and disturbance of HSP47 induces CHOP-dependent apoptosis[38].Thus, these results suggest two possible roles for klotho: klotho ameliorated ER stress and subsequently reduces the expression of GRP78 and HSP47 or klotho reduced the expression of GRP78 and HSP47 without inhibiting ER stress-induced apoptosis. Transcription factor CHOP is an important ER stress-mediated pro-apoptosis factor, which plays a critical role in myocardial apoptosis[14].We found that klotho reduced the expression of CHOP, therefore it was reasonable to assume that the reduction of ER chaperone proteins was due to ER stress inhibition. We also found that klotho inhibited ROS production in mouse heart and H9c2 cells. In brief, these data indicated that the anti-apoptosis effect of klotho was partially attributed to the amelioration of ER stress and oxidative stress. 

The MAPK pathway played important roles in cardiac apoptosis. Dominant-negative or constitutively activated forms of the p38 MAPK and ERK1/2 signaling pathways demonstrated that activation of p38 MAPK or inhibition of ERK1/2 were critical for induction of apoptosis in many cells, including cardiomyocytes[39]. Studies demonstrated that inhibition of ERK1/2 enhanced ischemia/reoxygenation–induced apoptosis and phenylephrine-induced hypertrophy in cardiomyocytes[40]. Inhibition of p38 MAPK and JNK efficiently attenuated cardiac remodeling[41]. Therefore, the dynamic balance between growth factor-activated ERK1/2 and stress-activated p38 and JNK is important in determining a cell’s fate. Evidence indicated the involvement of ERK1/2, p38 and JNK in ER stress-induced apoptosis in cardiomyocytes [42,43]. More recently, it was demonstrated that inhibition of p38 MAPK with the dominant-negative form efficiently reduced CHOP-mediated myocardial apoptosis[14]. In our study, SB203580 and SP600125 statistically inhibited ISO-induced ER stress and apoptosis, however, the PD98059 only slightly inhibited it. In accordance, klotho significantly inhibited ISO-induced phosphorylation of p38 and JNK, but did not remarkably effect the phosphorylation of ERK1/2 at each dose, indicating a better response to p38 and JNK . Therefore, it is reasonable to conclude that klotho suppresses apoptosis partially through inhibition of the p38 and JNK pathway. 

In conclusion, this study demonstrated that klotho effectively suppressed ISO-induced histological changes in mouse heart. Importantly, klotho played a pivotal role in inhibiting ISO-induced cardiomyocyte apoptosis in vivo and in vitro, and these effects were associated with amelioration of the ER stress-induced apoptosis signal through inhibition of the P38 and JNK pathway. In addition to uncovering a new action of klotho, these results could be clinically relevant for the treatment of heart failure. 

## Supporting Information

Figure S1 **Serum klotho concentration was increased by the administration of recombinant klotho.** Demonstration of the average serum concentration of klotho in CON, ISO, and ISO+KL groups. Data are mean±SEM, n=3. * P<0.05 between two compared groups; NS, no significance.
(TIF)

Figure S2 **Representative images of TUNEL assay in CON, ISO and ISO+KL groups at day9.** Nuclei of normal cells are blue, and nuclei of apoptosis cells are brown. Black arrows represent the positive staining of TUNEL. Scale bar=50 um, 400×.
(TIF)

Figure S3 **ISO significantly activated the phosphorylation of MAPK in a time-dependent manner.** (A) Representative images of western blotting of p-ERK1/2, ERK1/2, p-p38, p38, p-JNK and JNK in H9c2 cells at 0, 5, 15, 30 min after ISO treatment. (B,C,D) Demonstration of the phosphorylation of p-ERK1/2(B), p-p38(C) and p-JNK(D) at different times. Data are mean±SEM, n=3. * P<0.05 between two compared groups; NS, no significance.
(TIF)
