# Correction: Klotho Suppresses Cardiomyocyte Apoptosis in Mice with Stress-Induced Cardiac Injury via Downregulation of Endoplasmic Reticulum Stress

**DOI:** 10.1371/annotation/d7fe4fdf-4be4-4aa5-b3f0-d98790fc0b11

**Published:** 2013-12-31

**Authors:** Shuang Song, Pan Gao, Hang Xiao, Yan Xu, Liang Yi Si

The name of the last author is incorrect. The correct name is: Liang Yi Si.

