# Supplementary figures and images for: Klotho Suppresses Cardiomyocyte Apoptosis in Mice with Stress-Induced Cardiac Injury via Downregulation of Endoplasmic Reticulum Stress
Source: PLoS One. 2013 Dec 10;8(12):e82968. doi: 10.1371/journal.pone.0082968 (PMC3858310; doi:10.1371/journal.pone.0082968)

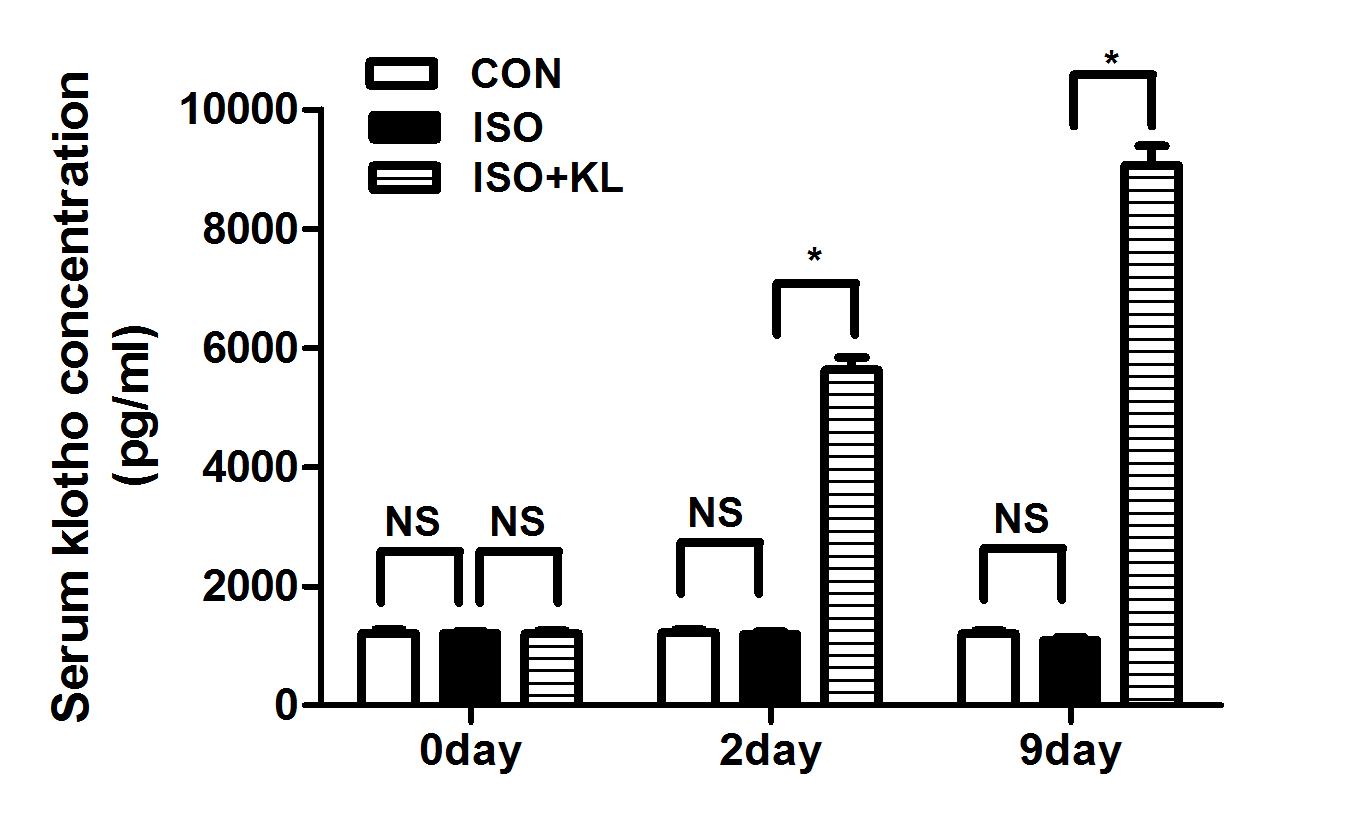

Supplement: Figure S1 — Serum klotho concentration was increased by the administration of recombinant klotho. Demonstration of the average serum concentration of klotho in CON, ISO, and ISO+KL groups. Data are mean±SEM, n=3. * P<0.05 between two compared groups; NS, no significance. (TIF) [file pone.0082968.s001.tif]

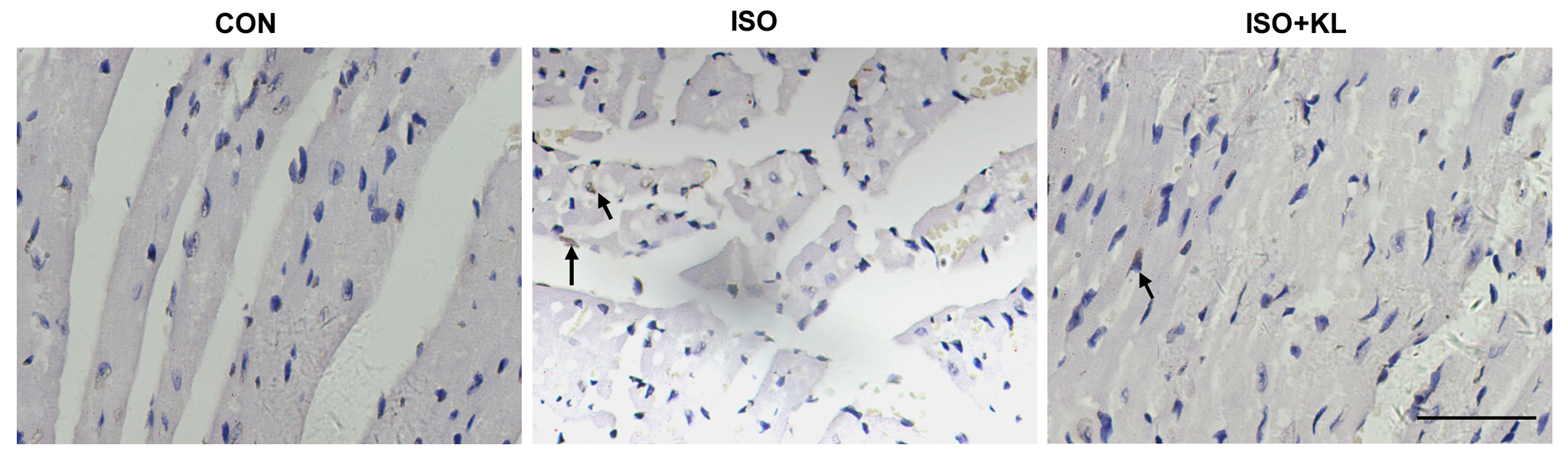

Supplement: Figure S2 — Representative images of TUNEL assay in CON, ISO and ISO+KL groups at day9. Nuclei of normal cells are blue, and nuclei of apoptosis cells are brown. Black arrows represent the positive staining of TUNEL. Scale bar=50 um, 400×. (TIF) [file pone.0082968.s002.tif]

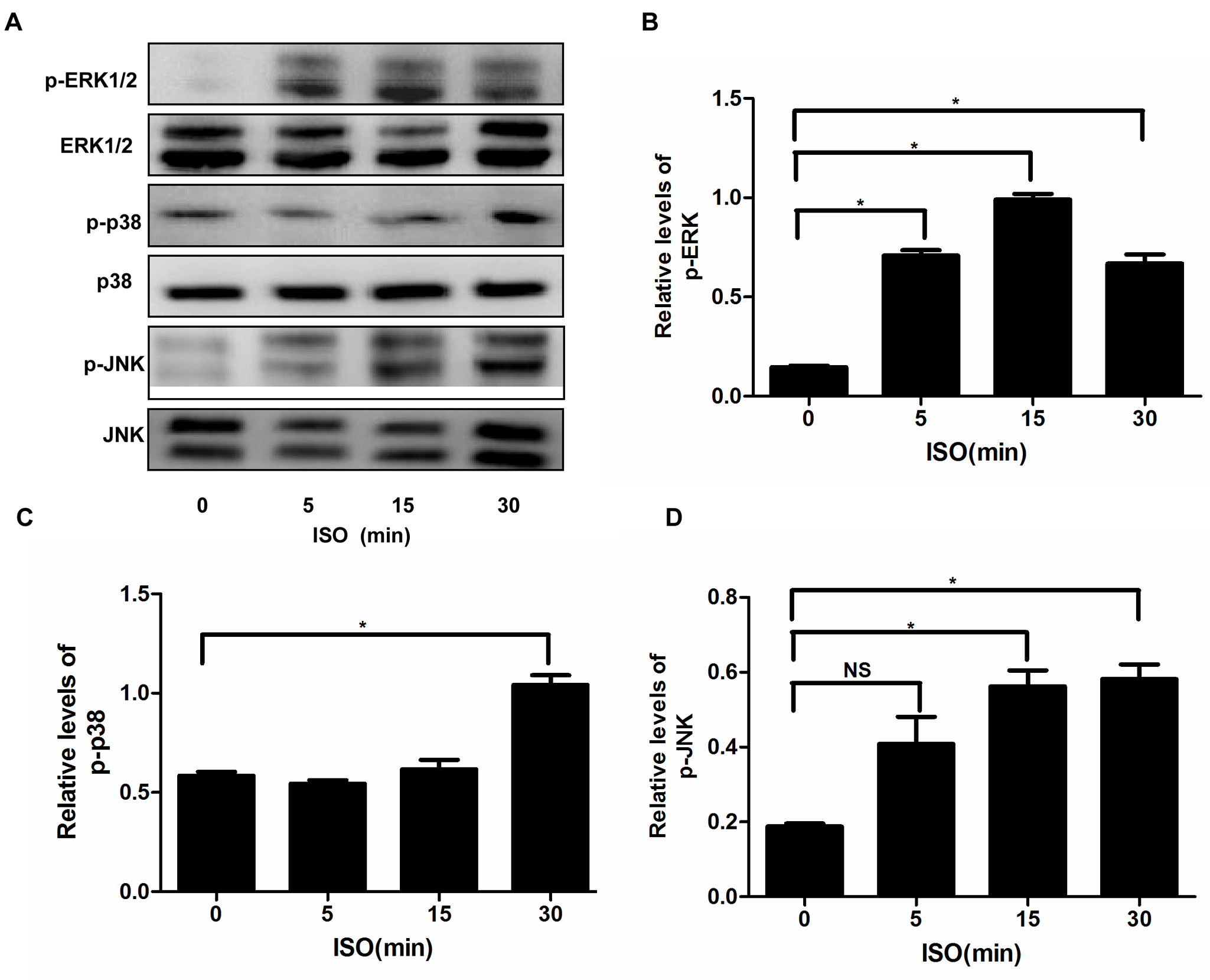

Supplement: Figure S3 — ISO significantly activated the phosphorylation of MAPK in a time-dependent manner. (A) Representative images of western blotting of p-ERK1/2, ERK1/2, p-p38, p38, p-JNK and JNK in H9c2 cells at 0, 5, 15, 30 min after ISO treatment. (B,C,D) Demonstration of the phosphorylation of p-ERK1/2(B), p-p38(C) and p-JNK(D) at different times. Data are mean±SEM, n=3. * P<0.05 between two compared groups; NS, no significance. (TIF) [file pone.0082968.s003.tif]
